# Solitäre lokoregionäre Metastase eines undifferenzierten pleomorphen Sarkoms im M. quadratus femoris

**DOI:** 10.1007/s00132-021-04093-w

**Published:** 2021-03-17

**Authors:** Mohamad Bdeir, Nikolaos Vassos, Ali Darwich, Cleo-Aron Weis, Sascha Gravius, Eva Renker

**Affiliations:** 1 Orthopädisch-Unfallchirurgisches Zentrum, Universitätsklinikum Mannheim, Medizinische Fakultät Mannheim der Universität Heidelberg, Theodor-Kutzer-Ufer 1–3, 68167 Mannheim, Deutschland; 2 Chirurgische Klinik, Universitätsklinikum Mannheim, Medizinische Fakultät Mannheim der Universität Heidelberg, Mannheim, Deutschland; 3 Pathologisches Institut, Universitätsklinikum Mannheim, Medizinische Fakultät Mannheim der Universität Heidelberg, Mannheim, Deutschland

**Keywords:** Weichgewebeneoplasie, Bestrahlung, Strahlentherapie, adjuvante, Muskelneoplasie, Onkologische Chirurgie, Soft tissue neoplasms, Radiation, Radiotherapy, adjuvant, Muscle neoplasms, Surgical oncology

## Abstract

Das undifferenzierte pleomorphe Sarkom („undifferentiated pleomorphic sarcoma“ [UPS]) gehört zur Gruppe der Weichteilsarkome und macht fast 10 % aller Weichteilsarkome aus. Der Fall eines 49-jährigen Patienten wird vorgestellt, bei dem die kompartmentorientierte Resektion eines primären UPS im linken Musculus gluteus maximus mit adjuvanter Radiotherapie (60 Gy) durchgeführt wurde. Im Rahmen der Tumornachsorge (3 Jahre später) wurde eine lokoregionäre Metastase an einer ungewöhnlichen Lokalisation im M. quadratus femoris festgestellt, welche mittels einer In-toto-Resektion mit intraoperativer Radiotherapie (10 Gy) behandelt wurde. Der intra- und postoperative Verlauf gestalten sich komplikationslos ohne neurologische Defizite. Im Rahmen der Nachtuntersuchung 6 Monate postoperativ war der Patient tumor- und beschwerdefrei.

## Abstract (en)

The undifferentiated pleomorphic sarcoma (UPS) is a part of the soft tissue sarcoma group and represents almost 10% of all soft tissue sarcomas. The case of a 49-year-old patient is presented who was diagnosed with a primary UPS in the left gluteus maximus muscle, which was treated with compartmental resection with adjuvant radiotherapy (60 Gy). During tumor follow-up (3 years later) a locoregional metastasis at an unusual location in the quadratus femoris muscle was detected, which was treated by in toto resection with intraoperative radiotherapy (10 Gy). The intra and postoperative outcome was without complications and without neurological deficits. In the last follow-up, 6 months postoperatively, the patient was free of tumors and symptoms.

## Anamnese

Die Erstvorstellung des damals 44-jährigen Patienten erfolgte vor 3 Jahren mit einer unklaren Raumforderung im M. gluteus maximus links. Nachdem eine auswärts durchgeführte sonographisch gesteuerte Stanzbiopsie keinen aussagekräftigen Befund erbracht hatte, wurde die Indikation zur primären Resektion gestellt. Es erfolgte eine kompartmentorientierte Resektion des Tumors. Die histopathologische Untersuchung ergab die Diagnose eines ca. 7 × 5 × 4,3 cm großen mäßig differenzierten pleomorphen Sarkoms (Grad 2 gemäß FNCLCC), welches R0-reseziert wurde (pT2a, G2, R0, Mx). Die Mitoserate betrug 3 Mitosen pro 10 HPF und die immunhistochemische Untersuchung ergab folgende Ergebnisse: Desmin positiv, Aktin positiv, CD56 teils positiv und CDK4 schwach positiv (S-100, CD34, Pan-Cytokeratin, MDM‑2 und EMA negativ) sowie eine niedrige Proliferationsrate (Ki67 von 5 %). Der postoperative Verlauf gestaltete sich komplikationslos. Bei Vorliegen einer mäßigen Differenzierung wurde die Indikation zur adjuvanten Radiotherapie gestellt, welche im Bereich der linken Glutealregion mit einer Gesamtdosis von 60 Gy durchgeführt wurde. Die weitere Tumornachsorge mittels Becken-MRT alle 3–6 Monaten und Thorax-CT alle 6–12 Monaten ergab keinen Anhalt für Lokalrezidiv oder Metastasen. Als Vorerkrankung ist nur eine essenzielle Hypertonie bekannt.

## Befund und Diagnose

**Figure 1:**
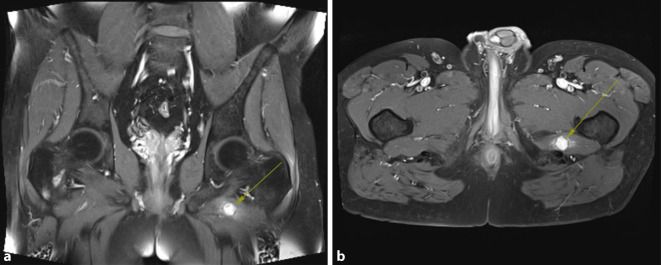

Im Rahmen der Tumornachsorge – 3 Jahre nach der initialen Operation – ließ sich kernspintomographisch eine verdächtige Raumforderung im Bereich des linken M. quadratus femoris darstellen (Abb. 1a, b). Eine Fernmetastasierung konnte computertomographisch ausgeschlossen werden. Bei gleicher MRT-Morphologie wie der Primärtumor mit deutlicher Hyperintensität in der T2- und Hypointensität in der T1-Wichtung sowie deutlichem Kontrastmittel-Enhancement bestand radiologisch der hochgradige V. a. eine lokoregionäre Metastase des pleomorphen Sarkoms, sodass nach einer interdisziplinären Fallbesprechung im Sarkomboard unseres zertifizierten Sarkomzentrums für Weichteilsarkome und Knochensarkome die Indikation zu einer erneuten Kompartmentresektion mit intraoperativer Radiotherapie ohne vorherige Biopsie gestellt wurde.

## Therapie und Verlauf

**Figure 2:**
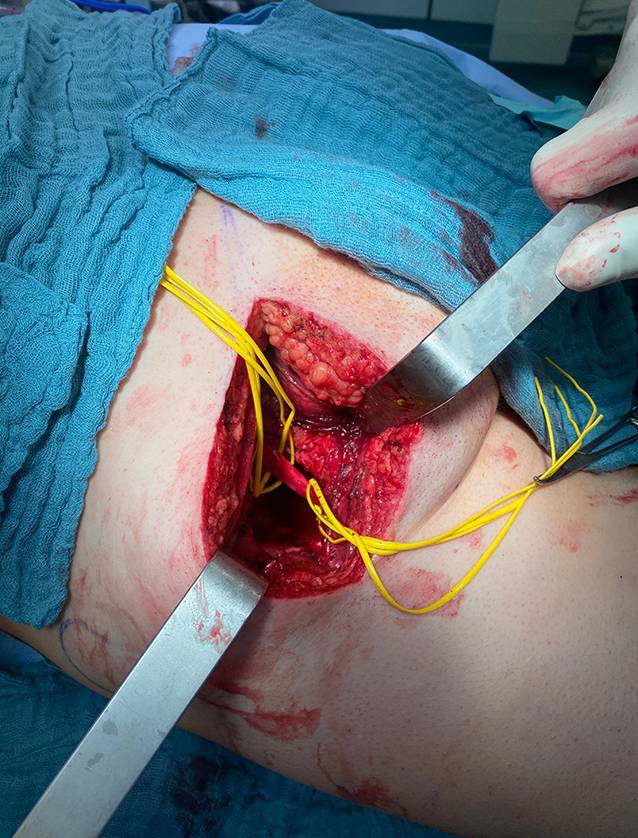

**Figure 3:**
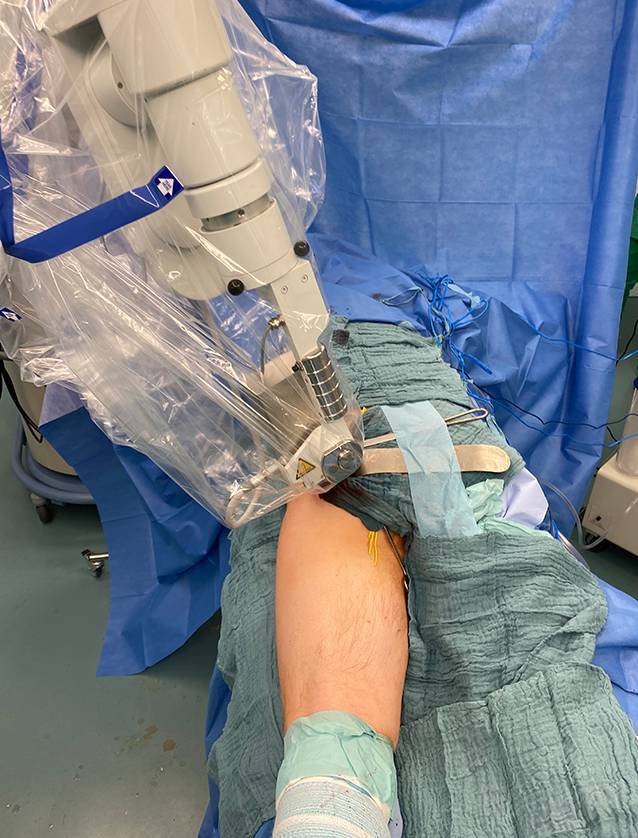

Der Patient wurde in Bauchlage in komplikationsloser Intubationsnarkose operiert. Intraoperativ wurde der N. ischiadicus freigelegt und angeschlungen (Abb. 2). Der Tumor wurde mit dem M. quadratus femoris in toto reseziert. Anschließend wurde eine intraoperative Bestrahlung des Tumorbetts mit insgesamt 10 Gy über 35 min durchgeführt (Abb. 3). Die Dauer der Operation und Bestrahlung betrug 3 h und 19 min, der Blutverlust etwa 100 ml. Der intra- und postoperative Verlauf gestaltete sich komplikationslos und der Patient konnte in gutem Allgemeinzustand am 4. postoperativen Tag entlassen werden.

**Figure 4:**
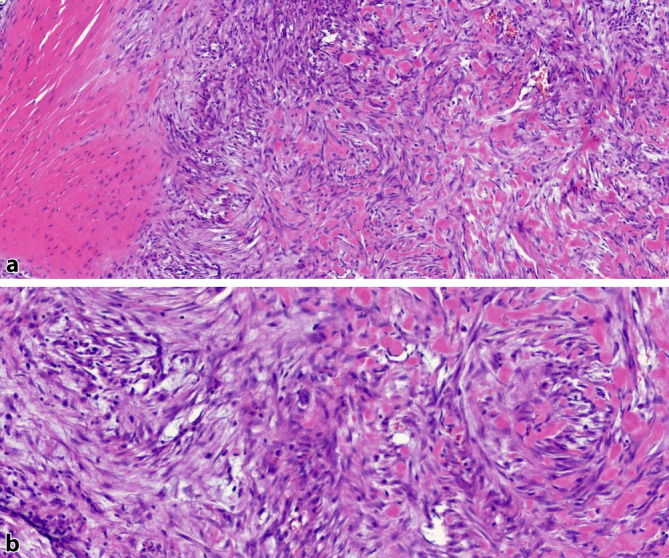

Makroskopisch maß das Weichteilresektat 8,5 × 6 × 4 cm, bestehend aus lipomatösen und muskulären Komponenten. Die histopathologische Untersuchung des Tumorherdes zeigte eine knotige, spindelzellige Tumorformation mit unregelmäßigen und pleomorphen Zellkernen (Abb. 4). Die Immunhistochemie fiel wie folgt aus: smAktin-positiv, CD56-positiv, p16-positiv, S100-negativ, Desmin-negativ, eine niedrig-mäßige Proliferationsrate (Ki67-Färbung: < 10). Der Befund war vereinbar mit einer lokoregionäre Metastase des vorbekannten undifferenzierten pleomorphen Sarkoms („undifferentiated pleomorphic sarcoma“ [UPS]) im M. quadratus femoris. Die Resektion erfolgte in sano (R0).

## Diskussion

Das UPS betrifft vor allem die Weichteile der Extremitäten und das Retroperitoneum mit einer höheren Inzidenz beim männlichen Geschlecht [10]. Die meisten Rezidive treten innerhalb von 2 Jahren nach der Erstbehandlung auf. Die lokale Rezidivrate nach Resektion wird in der Literatur mit 31–35 % [3, 5, 10] angegeben. In unserem Fall trat die lokoregionäre Metastase 3 Jahre nach der Erstbehandlung auf.

Zur Sicherung der Diagnose sollte in der Regel nach entsprechender bildgebender Beurteilung eine bioptische Sicherung erfolgen. Eine Exzisionsbiopsie kann für < 3 cm große oberflächliche Tumoren in Betracht gezogen werden [4].

In dem vorgestellten Fall erfolgte im Rahmen der interdisziplinären Tumorboardvorstellung eine Diskussion der Möglichkeiten einer bioptischen Sicherung mittels Feinnadelbiopsie versus primäre Resektion. Aufgrund der Nähe zum Primärtumor und der kernspintomographischen Morphologie der neu aufgetretenen Raumforderung, welche sich seitens der Tumormatrix und des Kontrastmittel-Aufnahmeverhaltens sowie des Wachstumsverhaltens identisch zum Primärtumor darstellte, legte sich die fachradiologische Beurteilung bezüglich der Dignität fest. Es wurde daher eine vollständige Resektion der 1,5 × 1,5 cm messenden lokoregionären Metastase beschlossen und auf eine vorherige bioptische Sicherung verzichtet.

Die chirurgische Resektion einer einzelnen Weichteilmetastase im Bereich der Extremitäten in toto ist die Therapie der Wahl. Unser Patient unterzog sich daher einer operativen Resektion.

Bei primären hochgradigen (G2–3) Weichteilsarkomen > 5 cm stellt die vollständige Tumorresektion und die adjuvante Strahlentherapie (prä- oder postoperativ) die Standardbehandlung dar [2, 13, 17]. Handelt es sich um ein niedriggradiges, tiefes Sarkom größer als 5 cm, kann eine Strahlentherapie unter Berücksichtigung der Histologie, der anatomischen Lage und der damit verbundenen Nebenwirkungen nach interdisziplinärer Besprechung erfolgen [4]. Zusätzlich wird in der Literatur der Nutzen einer adjuvanten Chemotherapie für eine Verlängerung des tumorfreien Intervalls (Auftreten von Fernmetastasen und Lokalrezidiven) bei Hochrisikopatienten (hochgradiger, tief gelegener Tumor und größer als 5 cm) diskutiert [6, 16]. Eine Metaanalyse bestätigte die marginale Wirksamkeit der Chemotherapie bei lokalisierten resektablen Weichteilsarkomen und fand einen Nutzen in Bezug auf das rezidivfreie Überleben und das Gesamtüberleben [11]. In den aktuellen Leitlinien stellt die adjuvante Chemotherapie allerdings keine Standardbehandlung dar und sollte nur als Therapieoption für einzelne Hochrisikopatienten in Betracht gezogen werden [4]. Eine neoadjuvante Chemotherapie mit Anthrazyklinen plus Ifosfamid für mindestens 3 Zyklen wird ebenso als eine Therapieoption für einzelne Hochrisikopatienten beschrieben [4].

Die etablierte intraoperative Strahlentherapie (IORT) wird in der Regel in einer Dosis von 10–15 Gy während der Operation mit Elektronen [7] oder als hochdosierte intraoperative Brachytherapie verabreicht [1]. In unserem Fall wurde die IORT über 35 min mit einer Gesamtdosis von 10 Gy appliziert. Die IORT eignet sich im Rezidivfall nach vorausgegangener Bestrahlung als Adjuvanz, da vorbestrahlte Haut- und andere Gewebestrukturen geschont werden können. Sie ist besonders vorteilhaft bei retroperitonealen und pelvinen Sarkomen, dennoch schränken die Toleranzen des umgebenden Normalgewebes die Dosis ein, die lokal appliziert werden kann. Es bleibt jedoch eine wirksame Strategie, da strahlenempfindliches Normalgewebe aus dem Bestrahlungsfeld herausgehalten werden kann. Wenn Normalgewebe wie Nerven, innere Organe oder Harnleiter dem Tumor anhaften und nicht aus dem Bestrahlungsfeld gehalten werden können, kann die IORT eine Verletzung dieser Strukturen verursachen. In solchen Fällen wird die Strahlendosis reduziert. Die Neuropathie ist die Hauptkomplikation, über die bei der IORT berichtet wird, und tritt bei etwa 10 % der Patienten auf. Bei Patienten mit retroperitonealen und pelvinen Sarkomen, die mit aggressiver Chirurgie, externer Bestrahlung und IORT behandelt wurden, wurden auch Fisteln und Ureterverletzungen beobachtet [7, 12]. Unser Patient hatte postoperativ keine Beschwerden oder neurologische Defizite. Die Nachuntersuchung mittels MRT vom Becken 6 Monaten postoperativ ergab keinen Anhalt für Lokalrezidiv oder Metastasen.

In der Literatur wurden verschiedene Lokalisationen des UPS wie z. B. pulmonal [14], im Pankreas [9], am Herzen [15] und in der Harnblase [8] beschrieben. Eine solitäre lokoregionäre Metastase des UPS im M. quadratus femoris, wie in dem vorgestellten Fall, stellt eine Besonderheit dar und wird zum ersten Mal beschrieben.

## Fazit für die Praxis

- Die regelmäßigen klinischen und radiologischen Verlaufskontrollen sind unabdingbar nach Erstdiagnose eines undifferenzierten pleomorphen Sarkoms unabhängig von der Art der Erstbehandlung.
- Die Therapie solcher Erkrankungen erfordert immer eine interdisziplinäre Zusammenarbeit erfahrener Fachdisziplinen in spezialisierten zertifizierten Sarkomzentren, um die bestmöglichen Ergebnisse für den Patienten mit dem entsprechenden Outcome zu erzielen.
- Trotz der unmittelbaren Nähe des N. ischiadicus zum Tumor im M. quadratus femoris konnte die intraoperative Radiotherapie ohne jegliche neurologischen Defizite erfolgen.

## Abkürzungen

CD: „Cluster of differentiation“
CDK: Cyclin-abhängige Kinase
EMA: Epitheliales Membranantigen
FNCLCC: Fédération Nationale des Centres de Lutte Contre le Cancer
HE: Hämatoxylin-Eosin
HPF: „High-power field“
IORT: Intraoperative Radiotherapie
MDM‑2: „Mouse double minute 2 homolog“
UPS: „Undifferentiated pleomorphic sarcoma“

## References

[1] Alektiar KM, Hu K, Anderson L (2000). High-dose-rate intraoperative radiation therapy (HDR-IORT) for retroperitoneal sarcomas. Int J Radiat Oncol Biol Phys.

[2] Beane JD, Yang JC, White D (2014). Efficacy of adjuvant radiation therapy in the treatment of soft tissue sarcoma of the extremity: 20-year follow-up of a randomized prospective trial. Ann Surg Oncol.

[3] Belal A, Kandil A, Allam A (2002). Malignant fibrous histiocytoma: a retrospective study of 109 cases. Am J Clin Oncol.

[4] Casali PG, Abecassis N, Aro HT (2018). Soft tissue and visceral sarcomas: ESMO-EURACAN Clinical Practice Guidelines for diagnosis, treatment and follow-up. Ann Oncol.

[5] Fletcher CD, Gustafson P, Rydholm A (2001). Clinicopathologic re-evaluation of 100 malignant fibrous histiocytomas: prognostic relevance of subclassification. J Clin Oncol.

[6] Frustaci S, Gherlinzoni F, De Paoli A (2001). Adjuvant chemotherapy for adult soft tissue sarcomas of the extremities and girdles: results of the Italian randomized cooperative trial. J Clin Oncol.

[7] Gieschen HL, Spiro IJ, Suit HD (2001). Long-term results of intraoperative electron beam radiotherapy for primary and recurrent retroperitoneal soft tissue sarcoma. Int J Radiat Oncol Biol Phys.

[8] Mylarappa P, Prathvi TJ (2013). Pleomorphic undifferentiated sarcoma of urinary bladder with calcified pulmonary metastasis: a rare entity. Indian J Urol.

[9] Ohsawa M, Mikuriya Y, Ohta K (2020). Rare pancreatic metastasis of undifferentiated pleomorphic sarcoma originating from the pelvis: a case report. Int J Surg Case Rep.

[10] Ozkurt B, Basarir K, Yildiz YH (2016). Primary malignant fibrous histiocytoma of long bones: long-term follow-up. Eklem Hastalik Cerrahisi.

[11] Pervaiz N, Colterjohn N, Farrokhyar F (2008). A systematic meta-analysis of randomized controlled trials of adjuvant chemotherapy for localized resectable soft-tissue sarcoma. Cancer.

[12] Petersen IA, Haddock MG, Donohue JH (2002). Use of intraoperative electron beam radiotherapy in the management of retroperitoneal soft tissue sarcomas. Int J Radiat Oncol Biol Phys.

[13] Pisters PW, Harrison LB, Leung DH (1996). Long-term results of a prospective randomized trial of adjuvant brachytherapy in soft tissue sarcoma. J Clin Oncol.

[14] Qorbani A, Nelson SD (2019). Primary pulmonary undifferentiated pleomorphic sarcoma (PPUPS). Autops Case Rep.

[15] Wilson TG, Jenkins P, Hoschtitzky A (2016). An extremely rare case of a high-grade pleomorphic cardiac sarcoma and likely cerebral metastasis in a young patient. Ecancermedicalscience.

[16] Woll PJ, Reichardt P, Le Cesne A (2012). Adjuvant chemotherapy with doxorubicin, ifosfamide, and lenograstim for resected soft-tissue sarcoma (EORTC 62931): a multicentre randomised controlled trial. Lancet Oncol.

[17] Yang JC, Chang AE, Baker AR (1998). Randomized prospective study of the benefit of adjuvant radiation therapy in the treatment of soft tissue sarcomas of the extremity. J Clin Oncol.

